# Characteristics of university students supported by counseling services: Analysis of psychological tests and pulse rate variability

**DOI:** 10.1371/journal.pone.0218357

**Published:** 2020-08-21

**Authors:** Yumi Adachi, Hiroaki Yoshikawa, Shigeru Yokoyama, Kazuo Iwasa

**Affiliations:** 1 Health Service Center, Kanazawa University, Kanazawa, Ishikawa, Japan; 2 Research Center for Child Mental Development, Kanazawa University, Kanazawa, Ishikawa, Japan; 3 Department of Neurology and Neurobiology of Ageing, Kanazawa University, Kanazawa, Ishikawa, Japan; Iwate Medical University, JAPAN

## Abstract

**Objective:**

Mental health is an essential issue during adolescence. The number of students who use counseling services is increasing in universities. We attempted to confirm the characteristics of the students who access counseling services using both psychological tests and pulse rate variability (PRV) for better support for students’ academic success.

**Methods:**

We recruited the participants for this study from the students who had counseling sessions at Kanazawa University (Group S). As a control group, we also recruited students who had no experience in counseling services (Group H). We obtained health information from the database of annual health checkups. Participants received the Wechsler Adult Intelligence Scale (WAIS)-III, Autism-Spectrum Quotient (AQ), Sukemune-Hiew (S-H) Resilience Test, and State-Trait Anxiety Inventory-JYZ (STAI). We also studied the 12-Item Short-Form Health Survey (SF-12v2) for testing Health-Related Quality of Life (HRQOL). As a physiological test, we examined the spectral analyses of pulse rate variability (PRV) by accelerating plethysmography. We performed a linear analysis of PRV for low-frequency power (LF: 0.02–0.15 Hz) and high-frequency power (HF: 0.15–0.50 Hz). We also conducted a non-linear analysis of PRV for the largest Lyapunov exponent (LLE). Additionally, we examined participants’ blood for autoantibodies against glutamate decarboxylase (GAD) 65.

**Results:**

A total of 105 students participated in this study. Group S had 37 participants (Male: 26, Female: 11), and Group H had 68 participants (Male: 27, Female 41). There were five males and one female in Group S who had diagnoses of autism spectrum disorder (ASD), and three males in Group S were diagnosed with attention deficit hyperactivity disorder (ADHD) by medical institutes. Additionally, four males and two females in Group S had diagnoses of ASD with ADHD by medical institutes. A male with ASD in Group S had epilepsy. The students of Group S had characteristics as follows: 1) lower power of Working Memory Index (WMI) despite high Full-Scale Intelligent Quotient (FSIQ), 2) higher ASD traits especially in Male, 3) lower resilience powers, 4) higher anxiety trait, 5) lower Health-Related Quality of Life (HRQOL) in Role/social component in both Male and Female, 6) lower HRQOL in Mental component in Male 7) shifting of autonomic nervous balance toward higher sympathetic activity.

**Conclusion:**

We could confirm the characteristics of students who visited counseling rooms for mental support (Group S). We also found gender differences in specificities of Group S. The educational system is changing rapidly to adjust social requests. These changes make conflict with the features of students of Group S. We should think about appropriate supports for the students who would pioneer the future of humanity.

## Introduction

Mental health is an essential issue during adolescence. Patel et al. reported that most mental disorders began during youth (12–24 years of age), although they are often first detected late in life [1]. The deterioration of mental health in adolescence sometimes resulted in suicide attempts. The study of global patterns of mortality in young people by Patton et al. reported that suicide is six % of all deaths in adolescence, and the mortality rate in youth is high in low-income and middle-income countries [2]. Adolescent-specific suicide prevention strategies have been implemented in three principal settings: schools, the community, and the health system [3]. Calear et al. did a systematic review of psychosocial suicide prevention interventions for youth [4]. They reported that the development of universal school-based interventions is promising.

In universities, students can receive various health services from health service facilities inside the universities. The health service facilities have physicians, licensed counselors, and nurses. Licensed counselors provide counseling services, as requested. The number of students seeking counseling services is increasing year by year, and their concerns are getting severe [5, 6]. Besides, a considerable number of students with autism spectrum disorder (ASD) traits, who were not diagnosed with ASD during the high school period, enter universities recently. Their difficulties with socialization have been found to affect their academic success and overall wellbeing [7]. Occasionally, university life reveals students’ mild developmental disorders, such as when students live alone apart from their parents. It is often that faculty members brought them to counseling services because of the absence of classes or low-grade point average (GPA). In the counseling rooms of universities, licensed counselors assess students’ conditions from a psychological perspective. The most common issues in their complaints are difficulties in university life, which are usually reported by subjective dialogs. If counselers found the necessity of further assessments, they would refer students to physicians, psychiatrists, or medical services outside of universities. If the symptoms or concerns of students were not severe, counselors do not examine them with further psychological tests.

In this study, we attempted to evaluate the mental and physical conditions of students who had counseling sessions by comparing them with students who did not use counseling services. According to our clinical experiences at the health service center, the students who utilized counseling services have a trait of ASD. However, we did not examine them in detail by psychological tests. Kanai et al. reported the Wechsler Adult Intelligence Scale (WAIS)-III profiles of adults with ASD, high-functioning autism, and high-functioning pervasive developmental disorders (PPD) [8]. It suggests that the WAIS-III is useful to evaluate the students who visit counseling rooms. Besides, the students who utilize the counseling rooms frequently complain of embodied symptoms such as hyperhidrosis, insomnia, and irritable bowels. They might be related to the dysregulation of autonomic nerves. Elam-Stock et al. reported that autonomic processing altered in ASD by using non-specific skin conductance response, which was an objective index of sympathetic neural activities [9]. For that reason, We tested autonomic nervous functions by spectral analyses of pulse rate variability (PRV) measured by accelerating plethysmography to evaluate autonomic nervous functions. We also assessed non-linear analyses of the pulse wave (largest Lyapunov exponent, LLE) recorded from the subjects’ fingertips that were related to the central nervous system function [10]. From our clinical experiences, the students who visited counseling services had lower resilience, higher anxieties, and a lower sense of the quality of life (QOL). So we assessed them with corresponding psychological tests. As a biological marker for ASD and attention deficit hyperactivity disorder (ADHD), we tested for the presence of auto-antibodies against glutamate decarboxylase (GAD65), which Rout et al. detected in 15% of ASD and 27% of ADHD children [11]. It is one of the neuronal antibodies detected in Stiff-person syndrome [12].

It is useful to determine the characteristics of the students who have distress in the university because we can offer them appropriate supports.

## Materials and methods

### Study design

This study is part of a randomized, cross-over, placebo-controlled trial (http://www.umin.ac.jp/ctr/index.htm, number UMIN000019101) in a single-center (Kanazawa University, Kanazawa, Japan). Participants were recruited from students of Kanazawa University. The study was conducted with Good Clinical Practice. Enrolment started in October 2015, and the last participant finished their observational period in October 2016. This report is a summary of the pre-evaluation of participants before entering the placebo-controlled trial.

### Standard protocol approvals, registrations, and participant consents

The Ethics Committee of Medicine, Kanazawa University approved the study (No. 29–3), and we obtained written informed consent from all subjects enrolled.

### Participants

We recruited participants from students of Kanazawa University (https://www.kanazawa-u.ac.jp/e/). We included students of Undergraduate and Graduate Schools of Humanities, Science and Technology and, Medicine, Pharmacy, and Health. Group S consisted of students who had counseling sessions in our counseling rooms. The students came to the counseling rooms by their own will or following the advice of friends or supervisors. Group H consisted of students recruited from classes who had no experience in counseling sessions at the university as well as outside of the university. We checked the medical and psychological records of participants candidates from the data of annual health checkups. Also, we had interview sessions with participants candidates for utilizations of the counseling sessions or psychiatric services outside the university. As exclusion criteria, we did not include students who had severe mental disorders attempting self-injury or suicidal attempt in this study. We are planning a study of a continuous response variable from independent control and experimental subjects with 2 controls per experimental subject. In a previous study [8], the results within each subject group were normally distributed with a standard deviation of 17. If the true difference in the experimental and control means is 10, we will need to study 35 experimental subjects and 70 control subjects to be able to reject the null hypothesis that the population means of the experimental and control groups are equal with probability (power) 0.8. The Type I error probability associated with this test of this null hypothesis is 0.05.

### Assessment

We interviewed candidates and screened their eligibility for this study. We obtained full informed consent using printed materials. We accessed health information on the participants from the database of annual health checkups in the Health Service Center, Kanazawa University. Participants received the WAIS-III, Autism-Spectrum Quotient (AQ), Sukemune-Hiew (S-H) Resilience Test, and State-Trait Anxiety Inventory-JYZ (STAI). We also studied the 12-Item Short-Form Health Survey (SF-12v2) (iHope International Co. Ltd., Kyoto, Japan) for testing Health-Related Quality of Life (HRQOL).

To evaluate the function of the autonomic nervous system, we investigated the spectral analyses of PRV measured by accelerating plethysmography. We performed a linear analysis of PRV for low-frequency power (LF: 0.02–0.15 Hz) and high-frequency power (HF: 0.15–0.50 Hz). We considered HF as an index of parasympathetic nervous tone and the ratio of LF to HF power (LF/HF) as an index of sympathetic nervous tone. We also performed a non-linear analysis of the data from the pulse wave study and obtained the values of the largest Lyapunov exponent (LLE). To study autoantibodies against GAD65, we drew 5 mL of blood from participants. The sera were separated and then stored at -80°C until antibody testing. The order of the assessments was as follows: 1) SF-12v2, 2) STAI, 3) S-H Resilience Test, 4) AQ, 5) PRV, 6) blood drawing, and 7) WAIS-III. The WAIS-III was performed on a different day from the other tests because of time constraints.

#### WAIS-III

The WAIS-III was developed to evaluate the Intelligent Quotient (IQ) in 1997. The version of WAIS-III used in this experiment was the Japanese version purchased from Nihon Bunka Kagakusha (Tokyo, Japan). The original version is published by Pearson (USA). Licensed psychologists performed the test. WAIS-III could obtain the Full-Scale Intelligence Quotient (FSIQ). FSIQ divided into Verbal IQ (VIQ) and Performance IQ (PIQ). VIQ contains Verbal Comprehension Index (VCI) and Working Memory Index (WMI). PIQ contains the Perceptual Organization Index (POI) and Processing Speed Index (PSI). The VCI includes the following tests: Vocabulary, Similarities, and Information. The WMI includes Arithmetic, Digit Span, and Letter-Number Sequencing. The POI includes Picture Completion, Block Design, and Matrix Reasoning. The PSI includes Digit Symbol-Coding and Symbol Search. Picture Arrangement, Comprehension, and Object Assembly are not used in the calculation of the Indexes.

#### AQ

The definition of AQ used in this experiment was based on the Japanese version of the original by Baron-Cohen *et al*. [13], which was revised in 2016. AQ has five subcategories: 1) Social skill (for example: ‘‘I prefer to do things with others rather than on my own.” -reversal item); 2) Attention switching (for example: ‘‘I prefer to do things the same way over and over again”); 3) Attention to detail (for example: ‘‘I often notice small sounds when others do not.”); 4) Communication (for example: ‘‘Other people frequently tell me that what I’ve said is impolite, even though I think it is polite.”); 5) Imagination (for example: ‘‘When I’m reading a story, I can easily imagine what the characters might look like.” -reversal item). The maximum score for each subcategory is 10. The total score is out of 50, and ASD is suspected when the score is equal to or more than 33.

#### S-H Resilience test

S-H Resilience Test was developed and validated by Sato and Sukemune [14] to evaluate the power of resilience in adults. We purchased printed test sheets from Takei Scientific Instruments Co., Ltd. (Niigata, Japan).

#### STAI

STAI was initially developed by Spielberger *et al*. [15], and the Japanese version (STAI-JYZ) was made by Hidano *et al*. (Jitsumu Kyoiku Shuppan, 2000). We purchased printed test sheets from Jitsumu Kyoiku Shuppan (Tokyo, Japan).

#### SF-12v2 Health Survey

To evaluate health-related quality of life (HRQOL), we used the SF-12v2 Health Survey (iHope International Co. Ltd., Kyoto, Japan). We evaluated lower measures of SF-12v2, which were composed of Physical Functioning (PF), Role Physical (RP), Bodily Pain (BP), General Health (GH), Vitality (VT), Social Functioning (SF), Role Emotional (RE), and Mental Health (MH). Then we transformed the scores from these summaries into Physical component summary (PCS), Mental component summary (MCS), and Role/Social component summary (RCS) scores [16].

#### Analyses of PRV

We performed spectral analyses of PRV measured by acceleration plethysmography. We collected data with an Android™ tablet installed with Alys™ (Chaos Technology Research Laboratory, Otsu, Japan) at a stable temperature and under quiet conditions. Participants sat on chairs and were asked to relax for five minutes before recording. We used fingertip sensors to record heart rates. If the recorded waves were too small to analyze correctly, we used sensors attached to the subjects’ earlobes. We recorded the data with the participant in a sitting position for three minutes. The collected data was calculated using Lyspect™ (Chaos Technology Research Laboratory, Otsu, Japan) installed in a PC with Windows 10. For spectral analyses, we used a fast Fourier transform (FFT). We set a low-frequency (LF) range from 0.04 to 0.15 Hz, and a high-frequency (HF) range from 0.15 to 0.40 Hz. The densities of the power spectrum were calculated from LF and HF, respectively, and the power ratio of LF/HF was obtained, which was estimated for the sympathetic activities. As an index of parasympathetic activities, we used the power spectrum of HF. We also performed a non-linear analysis of the pulse wave results and obtained LLE from the same data for the power spectrum.

#### Anti- GAD65 antibody

To measure anti-GAD65 antibody levels in serum, we used GADAb ELISA kits (Cosmic Corporation, Tokyo, Japan). Participants’ sera were tested using the instructions provided by the vendor.

### Statistical methods

For statistical analyses, we tested the data for normal distribution by the Shapiro-Wilk test. In the categories with a normal distribution, we analyzed data for equality of variance by two-tailed F test. For the data of equal variance, we utilized the Student t-test, For the data of non-equal variance, we used Welch's t-test. In the data with non-normal distribution, we utilized Wilcoxon-Mann-Whitney test (WMW). The findings were not accounted for multiple comparisons. We utilized JMP 14.3.0 (SAS Institute, Japan, Tokyo, Japan) for statistical analysis. We chose the acceptance level of significance as p<0.05.

## Results

### Participant disposition

A total of 105 participants were enrolled in this study. The number of Group S was 37, and that of Group H was 68 (Table 1). The actual number of students who visited our counseling room was 279 from April 2016 to March 2017 [17] and was 388 from April 2017 to March 2018 [18]. The total number of students at Kanazawa University of the fiscal year 2016 was 10488 (Undergraduate; 7895, Graduate and others; 2593). We obtained participants’ information on age and health information from the database of annual health checkups in April. As a result, age was higher in Group S than Group H in both Male and Female. Bodyweight (BW) was higher in Group S than Group H in Male. There were five males and one female who had a diagnosis of ASD, and three males and no female with ADHD by psychiatric services outside the university in Group S. Additionally, four males and two females had diagnoses of ASD with ADHD by psychiatric services outside the university in Group S. A male with ASD in Group S had epilepsy. In Male of Group S, three had a diagnosis of depression, one with bipolar disorder by psychiatric services outside the university. In Female of Group S, two had a diagnosis of bipolar disorder by psychiatric services outside the university. The race of participants was all Japanese. There was some familial background of mental disorders in Male of Group S.

**Table 1:** Clinical information of participants.

Male		S (N = 26)	H (N = 27)		p
	Age [median (IQR)]	22 (21–24) (min: 18, max: 30)	20 (19–21) (min: 18, max: 27)	S>H	0.0003^a^
	Height (cm), (mean ± SD)	172.9 ± 5.9	170.7 ± 5.7		0.1656^b^
	BW (kg) [median (IQR)]	62 (61–73)	57 (56–62)	S>H	0.0069^a^
	BMI [median (IQR)]	21.5 (19.2–24.0)	20.3 (19.5–21.3)		0.0781^a^
	Year Classes				
	Undergraduate 1	3	1		
	2	2	2		
	3	5	7		
	4	1	4		
	Graduate (Master) 1	2	2		
	2	3	1		
	Graduate (Doctor) 1	1	1		
	2	0	0		
	3	0	0		
		0	0		
	Clinical Diagnosis by the psychiatric service outside the university	ASD: 5 (1 with Epilepsy), ADHD: 3, ASD+ADHD; 4, Depression; 3, Bipolar disorder; 1	none		
	Suspected ASD by Autism Spectrum Quotient (AQ) score (≧33)	8 (30.8%)	2 (7.4%)		
	Family Background	Japanese: ASD (mother); 1, ADHD (father & sister); 1, Depression (mother); 1, Depression (mother & sister); 1	Japanese:		
	Months using the counseling services [median (min-max)]	4 (1–35)	none		
Female		S (N = 11)	H (N = 41)		p
	Age [median (IQR)]	23 (20–27) (min: 18, max: 28)	20 (19–21) (min: 18, max: 27)	S>H	0.0021^a^
	Height (cm), (mean ± SD)	160.3 ± 5.7	160.5 ± 5.1		0.8932^a^
	BW (kg) [median (IQR)]	53 (48–57)	52 (47–55)		0.4723^a^
	BMI [median (IQR)]	20 (19–24)	20 (18–22)		0.4529^a^
	Year Classes				
	Undergraduate 1	2	1		
	2	0	8		
	3	1	1		
	4	3	0		
	Graduate (Master) 1	0	6		
	2	2	3		
	Graduate (Doctor) 1	2	2		
	2	1	1		
	3	0	1		
			0		
			0		
	Clinical Diagnosis by the psychiatric service outside the university	ASD: 1, ASD+ADHD: 2, Bipolar disorder; 2	none		
	Suspected ASD by Autism Spectrum Quotient (AQ) score (≧33)	1 (9.1%)	1 (2.4%)		
	Family Background	Japanese:	Japanese:		
	Months using the counseling services [median (min-max)]	5 (1–39)	none		

^a^WMW

^b^t-test.

ASD: Autism spectrum disorder, ADHD: Attention Deficit Hyperactivity Disorder.

### WAIS-III

The WAIS-III showed significant differences in WMI of Total. Group S had lower values of WMI compared to Group H (Table 2). However, the value itself of WMI from Group S was within a normal range.

**Table 2:** WAIS-III IQ and index.

		Group S	Group H		p
Male		n = 26	n = 27		
FSIQ	(mean ± SD)	115.4 ± 11.7	117.3 ± 6.8		0.4774^b^
VIQ	(mean ± SD)	120.3 ± 11.5	122.7 ± 8.2		0.3981^b^
PIQ	(mean ± SD)	106.2 ± 16.2	106.3 ± 8.2		0.9852^b^
VCI	(mean ± SD)	120.9 ± 12.2	117.8 ± 7.8		0.2652^b^
POI	(mean ± SD)	105.2 ± 16.4	107.7 ± 10.0		0.5020^b^
WMI	(mean ± SD)	108.6 ± 11.1	114.3 ± 13.5		0.1014^b^
PSI	[median (IQR)]	104 (94–114)	107 (100–113)		0.2247^a^
Female		n = 11	n = 41		
FSIQ	(mean ± SD)	116.4 ± 9.7	117.8 ± 9.2		0.6517^b^
VIQ	(mean ± SD)	119.4 ± 9.0	120.3 ± 9.0		0.7401^b^
PIQ	(mean ± SD)	108.9 ± 15.3	110.7 ± 10.7		0.6584^b^
VCI	(mean ± SD)	121.8 ± 6.9	117.3 ± 10.3		0.1799^b^
POI	(mean ± SD)	110.5 ± 13.4	108.3 ± 12.9		0.6298^b^
WMI	(mean ± SD)	100.6 ± 14.2	110.1 ± 10.1		0.0599^c^
PSI	(mean ± SD)	105.7 ± 18.4	109.8 ± 13.3		0.4072^b^
Total		n = 37	N = 68		
FSIQ	(mean ± SD)	115.7 ± 11.1	117.6 ± 8.3		0.3193^b^
VIQ	(mean ± SD)	120.1 ± 10.0	121.3 ± 8.7		0.5244^b^
PIQ	(mean ± SD)	107.0 ± 15.8	108.9 ± 9.9		0.4478^b^
VCI	(mean ± SD)	121.2 ± 10.8	117.5 ± 9.3		0.0704^b^
POI	(mean ± SD)	106.8 ± 15.6	108.1 ± 11.7		0.6296^b^
WMI	(mean ± SD)	106.2 ± 12.5	111.8 ± 11.7	S<H	0.0308^c^
PSI	[median (IQR)]	102 (94–115)	107 (102–118)		0.0633^a^

^a^WMW

^b^t-test

^c^Welch’s t-test.

FSIQ: Full-Scale IQ, VIQ; Verbal IQ, PIQ; Performance IQ, VCI; Verbal Comprehension Index, POI; Perceptual Organization Index, WMI; Working Memory Index, PSI; Processing Speed Index.

In the Verbal and Performance tasks, the value of Similarities was significantly higher in Group S than in Group H for Male (Table 3). Symbol Search was significantly lower in Group S than in Group H for Male. In Female, Picture Arrangement was higher in Group S than in Group H. As Total, Similarities was significantly higher in Group S than in Group H. LetterNumber Sequencing, and Symbol Search are significantly lower in Group S than in Group H.

**Table 3:** WAIS-III tasks.

		Group S	Group H		p
Male		n = 26	n = 27		
	Verbal				
	Vocabulary [median (IQR)]	16 (13–18)	14 (13–17)		0.8647^a^
	Similarities [median (IQR)]	14 (13–16)	13 (11–14)	S>H	0.0064^a^
	Information (mean ± SD)	12.3 ± 2.2	13.3 ± 2.3		0.1204^b^
	Comprehension [median (IQR)]	12 (10–14)	14 (10–16)		0.2203^a^
	Arithmetic [median (IQR)]	13 (10–14)	13 (11–13)		0.9200^a^
	Digit Span [median (IQR)]	15 (12–16)	15 (13–17)		0.3330^a^
	Letter-Number Sequencing (mean ± SD)	10.2 ± 2.4	10.9 ± 2.2		0.3048^b^
	Performance				
	Picture Arrangement (mean ± SD)	9.5 ± 3.8	10.1 ± 2.4		0.4852^b^
	Picture Completion (mean ± SD)	10.8 ± 4.0	11.5 ± 2.3		0.4782^b^
	Block Design (mean ± SD)	12.1 ± 3.1	12.2 ± 3.0		0.9340^b^
	Matrix Reasoning (mean ± SD)	11.0 ± 2.9	11.6 ± 2.5		0.4013^b^
	Digit Symbol Coding [median (IQR)]	11 (8–14)	9 (1–11)		0.0766^a^
	Symbol Search (mean ± SD)	10.1 ± 3.1	11.7 ± 2.3	S<H	0.0393^c^
	Object Assembly (mean ± SD)	10.0 ± 4.2	9.1 ± 3.5		0.4332^b^
Female		n = 11	n = 41		
	Verbal				
	Vocabulary [median (IQR)]	15 (14–17)	14 (13–16)		0.2058^a^
	Similarities [median (IQR)]	14 (14–15)	14 (13–15)		0.1350^a^
	Information (mean ± SD)	10.5 ± 2.8	12.0 ± 2.4		0.0950^b^
	Comprehension (mean ± SD)	10.6 ± 3.0	12.4 ± 2.4		0.0849^c^
	Arithmetic (mean ± SD)	12.0 ± 2.8	11.5 ± 2.4		0.5692^b^
	Digit Span [median (IQR)]	15 (14–18)	15 (14–17)		0.8119^a^
	Letter-Number Sequencing (mean ± SD)	9.4 ± 2.8	11.0 ± 2.2		0.0539^b^
	Performance				
	Picture Arrangement [median (IQR)]	12 (11–14)	11 (8–13)	S>H	0.0337^a^
	Picture Completion (mean ± SD)	11.5 ± 3.9	12.2 ± 2.5		0.4750^b^
	Block Design (mean ± SD)	11.9 ± 3.8	12.6 ± 3.0		0.5493^b^
	Matrix Reasoning [median (IQR)]	12 (10–14)	12 (10–13)		0.6652^a^
	Digit Symbol Coding [median (IQR)]	11 (7–12)	11 (9–13)		0.1497^a^
	Symbol Search (mean ± SD)	10.7 ± 3.0	11.6 ± 3.1		0.4542^b^
	Object Assembly (mean ± SD)	11.0 ± 3.1	10.4 ± 3.0		0.5804^b^
Total		n = 37	n = 68		
	Verbal				
	Vocabulary [median (IQR)]	16 (13–18)	14 (13–16)		0.2289^a^
	Similarities [median (IQR)]	14 (13–15)	13 (12–14)	S>H	0.0084^a^
	Information (mean ± SD)	11.8 ± 2.5	12.5 ± 2.5		0.1595^b^
	Comprehension [median (IQR)]	11 (9–14)	13 (10–15)		0.0503^a^
	Arithmetic [median (IQR)]	13 (10–14)	12 (10–13)		0.3909^a^
	Digit Span [median (IQR)]	15 (14–16)	15 (13–17)		0.2762^a^
	Letter-Number Sequencing [median (IQR)]	10 (8–11)	11 (10–13)	S<H	0.0366^a^
	Performance				
	Picture Arrangement [median (IQR)]	11 (8–14)	10 (8–12)		0.6320^a^
	Picture Completion [median (IQR)]	10 (9–15)	12 (10–13)		0.2239^a^
	Block Design (mean ± SD)	12.1 ± 3.3	12.4 ± 3.0		0.5723^b^
	Matrix Reasoning [median (IQR)]	12 (10–13)	12 (10–13)		0.8066^a^
	Digit Symbol Coding [median (IQR)]	11 (8–13)	11 (8–13)		0.9622^a^
	Symbol Search [median (IQR)]	10 (8–12)	12 (9–13)	S<H	0.0333^a^
	Object Assembly (mean ± SD)	10.3 ± 3.9	9.9 ± 3.2		0.6125^b^

^a^WMW

^b^t-test

^c^Welch’s t-test.

### AQ score

The AQ scores in Male and Female showed significant differences (Table 4). In Male, AQ total, and subcategories AQ1 (Social skill), AQ2 (Attention switching), AQ4 (Communication) were significantly higher in Group S. The results suggest that Male in Group S tended ASD. However, Female did not have a significant tendency for ASD. In Total, AQ total, and subcategories AQ1 (Social skill), AQ2 (Attention switching), AQ4 (Communication), AQ5 (Imagination) were significantly higher in Group S than Group H. The number of participants who were suspected of having ASD based on their AQ score (≥33) was nine (24.3%) in Group S and three (4.4%) in Group H.

**Table 4:** AQ scores of participants.

		Group S	Group H		p
Male		n = 26	n = 27		
	AQ total (mean ± SD)	27.3 ± 7.7	21.2 ± 7.7	S>H	0.0056^c^
	AQ 1 (mean ± SD)	6.5 ± 2.5	4.3 ± 2.6	S>H	0.0026^c^
	AQ 2 (mean ± SD)	6.2 ± 1.8	4.8 ± 2.1	S>H	0.0109^c^
	AQ 3 [median (IQR)]	5 (2–7)	4 (3–6)		0.7878^a^
	AQ 4 (mean ± SD)	5.2 ± 2.7	3.6 ± 2.2	S>H	0.0217^c^
	AQ 5 (mean ± SD)	5.0 ± 2.2	4.0 ± 2.3		0.1237^b^
Female		n = 11	n = 41		
	AQ total (mean±SD)	24.0 ± 7.2	20.2 ± 6.3		0.0897^b^
	AQ 1 [median (IQR)]	8 (3–8)	4 (2–7)		0.1064^a^
	AQ 2 (mean±SD)	5.4 ± 1.6	4.6 ± 1.8		0.1905^b^
	AQ 3(mean±SD)	4.5 ± 2.3	4.5 ± 2.2		0.9646^b^
	AQ 4 [median (IQR)]	5 (2–6)	3 (2–5)		0.2809^a^
	AQ 5 [median (IQR)]	3 (2–6)	3 (1–5)		0.3568^a^
Total		n = 37	n = 68		
	AQ total (mean±SD)	26.3 ± 7.6	20.6 ± 6.9	S>H	0.0003^c^
	AQ 1 [median (IQR)]	7 (5–8)	4 (2–6)	S>H	0.0004^a^
	AQ 2 [median (IQR)]	6 (5–7)	4 (3–6)	S>H	0.0015^a^
	AQ 3 [median (IQR)]	5 (2–7)	5 (3–6)		0.7351^a^
	AQ 4 [median (IQR)]	5 (3–7)	4 (2–5)	S>H	0.0104^a^
	AQ 5 [median (IQR)]	4 (3–6)	3 (2–5)	S>H	0.0127^a^

^a^WMW

^b^t-test

^c^Welch’s t-test.

AQ-1; Social skill, AQ-2; Attention switching, AQ-3; Attention to detail, AQ-4; Communication, AQ-5; Imagination.

### The results of the S-H Resilience test

The results of the S-H Resilience Test are summarized in Table 5. S-H Resilience Scores were significantly lower in Total, Male, and Female of Group S than Group H. It means that Group S had less resilience than Group H.

**Table 5:** S-H Resilience test.

	Group S	Group H		p
Male	n = 26	n = 27		
(mean ± SD)	86.8 ± 19.2	100.9 ± 14.3	S<H	0.0042^b^
Female	n = 11	n = 41		
(mean ± SD)	91.3 ± 10.1	103.9 ± 12.3	S<H	0.0025^b^
Total	n = 37	n = 68		
[median (IQR)]	88 (75–101)	103 (97–110)	S<H	<0.0001^a^

^a^WMW

^b^Welch’s t-test.

### The results of the STAI

We summarized the results of the STAI in Table 6. The State Anxiety Scores of Male and Total were significantly higher in Group S than in Group H, but Female did not. The Trait Anxiety Scores of Male, Female, and Total were significantly higher in Group S than Group H.

**Table 6:** STAI.

		Group S	Group H		p
Male		n = 26	n = 27		
	State Anxiety Score [median (IQR)]	43 (37–52)	38 (33–42)	S>H	0.0237^a^
	Trait Anxiety Score (mean ± SD)	53.6 ± 10.0	44.9 ± 10.5	S>H	0.0033^c^
Female		n = 11	n = 41		
	State Anxiety Score (mean ± SD)	42.0 ± 9.0	40.2 ± 8.8		0.5463^b^
	Trait Anxiety Score (mean ± SD)	57.2 ± 11.9	47.0 ± 9.8	S>H	0.0212^c^
Total		n = 37	n = 68		
	State Anxiety Score [median (IQR)]	42 (37–50)	39 (34–44)	S>H	0.0311^a^
	Trait Anxiety Score (mean ± SD)	54.7 ± 10.6	46.2 ± 10.1	S>H	0.0002^c^

^a^WMW

^b^t-test

^c^Welch’s t-test.

### SF12v2 Health Survey

We summarized the results of the SF12v2 Health Survey in Table 7. The Male, Female, and Total of Group S had significantly lower RCS scores than Group H. In Male, Group S had significantly lower MCS besides RCS than Group H. The results indicate Group S had lower HRQOL of Role/Social component. Additionally, males of Group S had lower HRQOL of Mental component. These results reflect the difficulties of Group S in college life.

**Table 7:** SF12v2 Health Survey.

		Group S	Group H		p
Male		n = 26	n = 27		
	PCS (mean ± SD)	58.3 ± 8.3	56.8 ± 4.6		0.4039^b^
	MCS [median (IQR)]	50 (41–54)	53 (49–58)	S<H	0.0434^a^
	RCS (mean ± SD)	38.3 ± 12.6	45.5 ± 10.7	S<H	0.0289^c^
Female		n = 11	n = 41		
	PCS [median (IQR)]	62 (58–65)	59 (55–63)		0.1717^a^
	MCS (mean ± SD)	48.7 ± 8.0	50.0 ± 8.1		0.6370^b^
	RCS (mean ± SD)	32.7 ± 9.0	43.0 ± 10.9	S<H	0.0045^c^
Total		n = 37	n = 68		
	PCS [median (IQR)]	59 (53–65)	58 (54–62)		0.4055^a^
	MCS [median (IQR)]	50 (43–54)	52 (47–56)		0.1149^a^
	RCS (mean ± SD)	36.6 ± 11.8	44.0 ± 10.8	S<H	0.0024^c^

^a^WMW

^b^t-test

^c^Welch’s t-test.

PCS; Physical component summary, MCS; Mental component summary, RCS; Role/Social component summary.

### Analyses of PRV

Linear and non-linear analyses of the pulse wave results were obtained by accelerating plethysmography. We collected the powers of LF and HF by spectral analysis of PRV. The ratio of LF/HF is estimated as sympathetic nervous tone, and HF is representative of activities of the parasympathetic nerve. The LF/HF was significantly higher in the Total of Group S than that of Group H. The HF was lower in Group S, but there was no significant difference. These findings mean that the activity of the autonomic nerve of Group S shifted toward the hyper sympathetic status (Table 8). In the analyses by genders, there was no difference between Group S and Group H. We considered it is because the data of pulse wave is variable. Non-linear analysis of PRV brought us the LLE of the attractor, which is constructed for the time series data from pulse waves. There was no significant difference in LLE, and LLE (SD) between Group S and Group H. The coefficient of variations of R-R intervals (CVRR %) were not different between Group S and Group H. The values of heart rate (HR) were not different between Group S and Group H.

**Table 8:** Analyses of PRV, CVRR, and HR.

	Group S	Group H		p
Male	n = 26	n = 27		
LF [median (IQR)]	10 (5–16)	7 (5–12)		0.4933^a^
HF [median (IQR)]	4 (2–9)	5 (3–9)		0.7964^a^
LF/HF [median (IQR)]	2 (1–3)	1 (1–3)		0.0998^a^
LLE (mean ± SD)	4.9 ± 2.1	5.4 ± 1.8		0.3216^b^
LLE (SD) [median (IQR)]	1 (1–2)	1 (1–2)		0.3595^a^
HR (mean ± SD)	75.1 ± 12.2	74.4 ± 8.9		0.8176^b^
CVRR [median (IQR)]	6 (4–9)	6 (5–7)		0.9787^a^
Female	n = 11	n = 39		
LF [median (IQR)]	8 (5–10)	8 (6–13)		0.6064^a^
HF [median (IQR)]	8 (4–12)	9 (4–13)		0.5901^a^
LF/HF [median (IQR)]	1 (1–2)	1 (1–2)		0.9254^a^
LLE (mean ± SD)	4.9 ± 2.1	4.8 ± 1.2		0.9357^b^
LLE (SD) (mean ± SD)	1.2 ± 0.4	1.1 ± 0.4		0.5363^b^
HR (mean ± SD)	74.0 ± 9.6	73.9 ± 8.9		0.9938^b^
CVRR [median (IQR)]	6 (5–8)	6 (5–7)		0.7430^a^
Total	n = 37	n = 66		
LF [median (IQR)]	10 (5–13)	8 (5–13)		0.8339^a^
HF [median (IQR)]	5 (3–9)	7 (4–10)		0.2468^a^
LF/HF [median (IQR)]	2 (1–3)	1 (1–2)	S>H	0.0382^a^
LLE [median (IQR)]	4 (4–6)	5 (4–6)		0.3808^a^
LLE (SD) [median (IQR)]	1 (1–2)	1 (1–1)		0.7545^a^
HR (mean ± SD)	74.7 ± 11.3	74.1 ± 8.8		0.7611^b^
CVRR [median (IQR)]	6 (5–9)	6 (5–7)		0.7966^a^

^a^WMW

^b^t-test.

LF; low frequency, FH; high frequency, LF/HF; low frequency/high frequency, LLE; largest Lyapunov exponent, LLE (SD); largest Lyapunov exponent (standard deviation), CVRR; coefficient of variation of R-R intervals.

### Anti-GAD65 antibody

We tested the level of anti-GAD65 antibody using the participants’ sera. The cutoff value settled at 5.0 U/mL. There were only three participants who had positive results, two from Group S (9.0 U/mL, 7.7 U/mL) and one from Group H (10.2 U/mL). The two participants with a positive result for anti-GAD65 antibody from Group S had a diagnosis of ADH. There was no overall difference in anti-GAD65 antibody titers between Group S and Group H (Table 9).

**Table 9:** Anti-GAD65 antibody.

Anti-GAD65 antibody	Group S	Group H	
(U/mL)	median (IQR)	median (IQR)	p
	n = 22	n = 22	
Male	1.6 (0.2–2.2)	1.2 (0.4–2.3)	0.6886^a^
	n = 8	n = 31	
Female	1.4 (0.1–3.6)	1.7 (0.2–3.2)	0.9029^a^
	n = 30	n = 53	
Total	1.7 (0.2–2.3)	1.4 (0.3–3.0)	0.9815^a^

^a^WMW.

## Discussion

The purpose of this study was to clarify the commonalities between students who utilize the counseling services of the university, especially interested in ASD features. The average age of Group S was significantly higher than Group H for both Male and Female (Table 1). BW was significantly higher in Male of Group S than those of Group H. The higher BW may be attributable to two participants who had a considerable BW. As far as accompanying conditions, 12/26 (46.2%) of males in Group S and 3/11 (27.3%) of females in Group S had a diagnosis of neurodevelopmental disorders by psychiatrists of medical services outside of the university.

WAIS-III revealed several characteristics of Group S. Group S (Total) had significantly lower scores of WMI than Group H (Total). (Table 2). However, the values of WMI in Group S were within the normal range. Regarding the WAIS-III Tasks, the score of Similarities was significantly higher in Group S than Group H in Male. The score of Symbol Search was significantly lower in Group S than Group H in Male. In Female, Picture Arrangement was significantly higher in Group S than Group H. As a result, Male and Female had different characteristics in WAIS-III results. These results indicate the inferiority of WMI of Group S. Working memory is a function whereby the person stores useful information in their mind for a short period, which typically decreases with age [19]. It is related to the functional connectivity of large scale brain networks [20]. From these findings, we have to consider the specificities of Group S to provide better maneuvers for the mental health of university students. Recently, the educational system of Japanese universities is changing drastically; for example, the introduction of classes taught by the English language, quarterly terms, requirement of skills in information and communication technology (ICT), and active learning strategies. Besides, studying abroad for several school terms is recommended. These changes require much higher abilities for students than ever.

The AQ score was significantly higher in the Male of Group S than that of Group H (Table 4). Therefore, The Male of Group S tends toward ASD. Regarding the results of the S-H Resilience test, Group S had significantly lower scores in both Male and Female (Table 5). Resilience links to academic success [21, 22]. As far as STAI, the Trait Anxiety Score was significantly higher in both Male and Female of Group S (Table 6). The high Trait Anxiety scores suggest that students in Group S were in a stable anxiety state.

The SF12v2 revealed that RCS was significantly lower in Group S than Group H in both Male and Female. The low scores in RCS mean that these students faced challenges pursuing necessary communication in campus life. Additionally, Male in Group S had significantly lower scores in MCS than Male in Group H. It means that Male of Group S had difficulties in mental health. The PCS in Group S in both Male and Female was not different from that of Group H. The participants were all in their twenties, and their physical status was in good condition. It may be the reason why no difference in PCS between Group S and Group H.

Spectrum analysis of PRV is useful for studying human brain functions. Previously, we indicated that patients with Lewy body dementia (DLB) had significantly lower LF/HF values compared to patients with Alzheimer's disease (AD) [23]. We also showed paradoxical parasympathetic nervous activities of migraine patients by the orthostatic load [24]. Spectrum analysis of PRV indicated that those subjects who scored highly in LF/H in Group S inclined to hyper sympathetic nervous status. HF, which was an indicator of parasympathetic activity, tended to be lower in Group S but not significantly different, even when we recorded the PRV in calm conditions after five minutes of rest. Our findings may support the dysfunction of parasympathetic activities. Porges et al. proposed the polyvagal theory [25, 26]. They suggested that patients with ASD had a malfunction of parasympathetic nervous activity, and it resulted in difficulties in communications. We need further study to connect our findings with the polyvagal theory. Some intervention to adjust the autonomic nerve toward parasympathetic may improve the problems faced by the subjects in Group S. Non-linear analysis of accelerated plethysmography (LLE) showed no difference between Group S and Group H. LLE is a useful indicator of mental health [10]. A low level of LLE indicates that the subject is unable to adapt to external problems, which is characteristic of dementia and depression sufferers. A continuous high level of LLE implies external adaptability. Recently, researchers began to think about brain function in the model of the functional brain network [27]. The functional brain network related to the state of rest was defined as the default mode network (DMN) [28, 29]. It is involved in the large scale brain network both during rest and cognitive tasks [30]. We suspect LLE may represent the brain activity and may have a relationship with DMN. The functional brain network is also related to working memory [31, 32]. Our results for WAIS-III indicate that Group S had significantly lower scores in WMI compared with Group H (Table 2). The presence of working memory deficits in high-functioning adolescents with ASD is disputed [33]. Chien et al. suggested the existence of deficient visuospatial working memory and corresponding neural correlates within the DMN in adolescents with ASD [34]. These specificities make academic life difficult for students of Group S and evoke mental distress.

Regarding the autoantibody against GAD65, we could not confirm the significance reported by Rout *et al*. [11]. Recently, there have been reports of an association between ASD and anti-glutamate NMDA receptor antibodies [35, 36]. The etiology of ASD is multi-factorial [37]. In further studies, we need to examine other autoantibodies against molecules related to brain function.

We admit that the limitation of our study is the shortness of sample size. The initial study plan did not expect the differences by gender. In the process of analysis, we noticed the meaningful differences by gender. Additionally, we could see several significant trends in the data, but statistically not different. To elucidate the characteristics by gender, we should perform the study on a larger scale. It requires us a study plan in a multicenter. We also admit that our analysis is limited because the data of pulse wave analysis is so variable. The pulse wave is an embodied mind; therefore, it fluctuates vigorously. We have to develop better methods to analyze the pulse wave, which represents the human mind.

In conclusion, we could confirm the specific characteristics of university students who visited counseling services. They are as follows: 1) lower power of WMI despite high FSIQ, 2) higher ASD traits especially in Male, 3) lower resilience powers, 4) higher anxiety trait, 5) lower QOL in Role/social component in both Male and Female, 6) lower HRQOL in Mental component in Male 7) shifting of autonomic nervous balance toward higher sympathetic activity. The educational system is changing rapidly to adjust social requests. These changes make conflict with the characteristics of students of Group S. We should think about appropriate supports for the students who would pioneer the future of humanity.
